# Functional Additives in Automotive Polymer Matrices: Compatibility, Mechanisms, and Industry Challenges

**DOI:** 10.3390/polym17172328

**Published:** 2025-08-28

**Authors:** Dan Dobrotă, Andreea-Mihaela Bărbușiu, Gabriela-Andreea Sava, Valentin Ștefan Oleksik

**Affiliations:** 1 Faculty of Engineering, Lucian Blaga University of Sibiu, 550024 Sibiu, Romania; andreea.barbusiu@ulbsibiu.ro (A.-M.B.); andreea.sava@ulbsibiu.ro (G.-A.S.); valentin.oleksik@ulbsibiu.ro (V.Ș.O.); 2 Academy of Romanian Scientists, Ilfov Street 3, 050045 Bucharest, Romania

**Keywords:** polymer matrix, additive, automotive, compatibility

## Abstract

This review supports formulation engineers in designing compatible and regulation-compliant additive systems. The integration of functional additives into polymer matrices plays a pivotal role in tailoring material properties to meet the demanding performance, safety, and sustainability criteria of the automotive industry. Key findings highlight that (1) optimal additive loadings are critical for balancing performance and mechanical integrity; (2) HALS and benzotriazole-based UV stabilizers extend service life by up to 3000 h in accelerated weathering without modulus loss; (3) bio-based plasticizers such as ESO and ATBC reduce migration rates by 30–40% compared to conventional phthalates; (4) phosphorus-based flame retardants and zinc borate synergistically achieve UL-94 V-0 ratings with minimal smoke release. This work introduces an integrative mapping of additive–polymer interactions under real-world conditions, coupled with synthesis tables that provide multi-criteria evaluations of performance, limitations, and sustainability—tools not present in prior literature. In contrast to previous reviews, this work introduces an integrative mapping of additive–polymer interactions under real-world automotive stressors, explicitly linking performance, compatibility, regulatory compliance, and sustainability. In addition, a series of synthesis consolidate multi-criteria evaluations—covering functional performance, technical limitations, regulatory risks, and sustainability potential—which provide practitioners with a decision-support tool not found in prior literature. These features constitute the primary methodological and practical contributions of this review. This review uniquely integrates an “evidence strength” assessment into synthesis tables and develops an integrative mapping of polymer–additive systems, offering actionable guidelines that go beyond prior literature reviews.

## 1. Introduction

**Figure 1:**
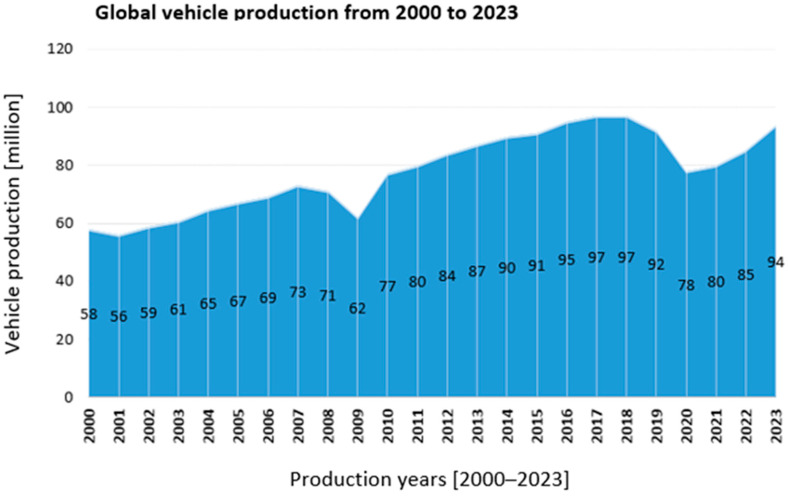
Global vehicle production from 2000 to 2023.

Global vehicle production increased from 58 million units in 2000 to 94 million in 2023, reflecting the sustained growth of the automotive sector and its economic significance (Figure 1). This expansion has intensified the demand for lightweight, high-performance polymer composites capable of meeting stringent mechanical, thermal, and environmental standards. However, the intrinsic properties of most polymer matrices require optimization through functional additives to ensure long-term performance under mechanical stress, temperature fluctuations, UV radiation, and chemical exposure. Functional additives enhance processing behavior, durability, and regulatory compliance, making them indispensable for replacing heavier materials such as steel and aluminum in automotive applications [1]. Recent data confirm that the upward trend in global vehicle production has continued in the post-pandemic period, with an average annual growth rate of 2.4% between 2020 and 2023, driven primarily by market expansion in Asia and Central Europe [2]. The continuous expansion of the automotive industry—reaching 94 million vehicles produced globally in 2023—has led to increasing demands for energy efficiency, cost reduction, and environmental compliance. One of the major strategies adopted to meet these challenges is the replacement of traditional metal parts with lightweight, high-performance polymer composites. However, the intrinsic properties of polymer matrices are often insufficient for meeting the complex technical and regulatory requirements of automotive applications. To ensure performance under mechanical stress, temperature fluctuations, UV radiation, and exposure to chemicals, polymers must be functionalized through the incorporation of additives.

Functional additives enhance processing behavior, mechanical durability, fire resistance, and longevity of plastic components. Although the literature offers substantial information on individual additives and their effects, there is a lack of integrative frameworks that correlate additive–polymer compatibility with real-world automotive conditions—including durability, toxicity, recyclability, and synergistic effects of additive combinations. Furthermore, legislative restrictions on hazardous compounds (e.g., phthalates, brominated flame retardants) and the push toward sustainable materials require a re-evaluation of current additive practices. This review aims to fill this gap by critically examining the mechanisms of action, compatibility factors, and industrial relevance of key functional additives used in automotive polymer systems.

While previous reviews have examined individual additive classes, none have integrated performance, regulatory, and sustainability criteria into a unified analytical framework as proposed in this work.

Overall, Figure 1 illustrates a long-term global increase in vehicle production, interrupted by occasional declines caused by global economic and health-related factors.

The increase in requirements also brings new requirements, including the reduction of carbon emissions. The use of polymeric materials in the car manufacturing industry has proven to be a reliable solution, respecting the imposed norms. However, the basic properties they possess are not sufficient for the automotive industry. The performance of plastics is optimized by adding additives to the chemical structure, which makes them capable of replacing heavy metals in cars [3].

Polymer composites offer a superior strength-to-weight ratio compared to structural steel, while also lowering costs associated with machining and corrosion protection. In the past, vehicles used approximately 1163 kg of iron and steel, and currently, these have been replaced by 150 kg of plastics and composites [3]. The demand for plastics in the automotive industry has reached approximately 4.3 Mt/year, approximately 8.6% of the total demand for plastics in Europe, which ranks the automotive sector in third place. The plastic components in a car represent between 15 and 18% of its total mass [4].

A 2024 Plastics Europe report corroborates these values, noting that the automotive sector remains among the top three consumers of polymers in the European Union, with a slight increase compared to the decade average [5].

The use of glass and carbon fibers in polymer reinforcement increases stiffness by 30–50% and enhances fatigue resistance, making them essential for components subjected to cyclic loading [6]. Polymeric materials have been highlighted by the physical and mechanical properties they possess, namely fatigue and impact resistance, low weight, high rigidity, and superior shock absorption resistance [5,6]. At the same time, due to their unique properties, polymer composites have the ability to replace metallic materials, such as steel or aluminum, to reduce the weight of components or products [7,8]. Polymers reinforced with glass or carbon fibers have significantly superior characteristics and contribute to reducing the weight of vehicles [9,10]. Minimizing the weight of a vehicle by 10% reduces fuel consumption and carbon emissions from 5% to 7% [3].

Similar results are reported in SAE studies, where a 100 kg reduction in vehicle mass leads to fuel savings of approximately 0.4 L/100 km under real-world operating conditions [8].

The sustainability of the automotive industry depends on the use of polymer composite materials in the design and manufacture of vehicles [2]. High-performance plastics offer designers a wide range of geometric and economic solutions in the construction of vehicle parts [3]. In a vehicle, there are over 35 types of polymers used depending on the four destinations of the components [4], as follows:

- Exterior (e.g., bumpers, headlights).
- Interior (e.g., dashboard, door panel).
- Under the hood (e.g., tanks, engine cover, cable insulation).
- Decorations (e.g., wheel covers, spoiler, wipers).

Depending on the purpose and role of the machine components, whether they are interior or exterior, the polymers used must meet the required technical requirements. The requirements include, among others, dimensional stability, resistance to mechanical tests, high temperatures, humidity, UV rays, chemical agents, and the aesthetic characteristics of the part.

Table 1 highlights a series of plastic components encountered in the construction of a car and the type of polymer matrix used in their manufacture. The polymer matrix is chosen according to technical, technological, and economic characteristics.

For example, polyethers (PBT, PPE) and polyamides are often found in the manufacture of under-the-hood components, due to their high resistance to heat and petroleum products. Polypropylene (PP) is a lightweight and adaptable material due to its balance between mechanical characteristics and low price, which is why it is used in dashboards and bodywork. ABS, PVC, or ASA are plastics used in interior design components due to their durability, visual appearance, and compatibility with color additives.

Plastics are obtained by polymerizing monomers, additives, and other substances [11]. In their pure form, these materials do not meet people’s needs for everyday applications, but also for specialized ones; for this reason, they need to be adapted using functional additives [12]. Currently, there are approximately 10,000 additives for plastics [13]. The role of additives is to improve the properties of the polymer, so that it is harder, wear-resistant, flexible, to protect against UV rays, and also to help in the manufacturing process [11]. They are found in both liquid and solid forms, such as powders, flakes, granules, spheres, emulsions, and beads. Manufacturing processes, such as extrusion, spraying, and grinding, define the final form of the additive [12].

**Figure 2:**
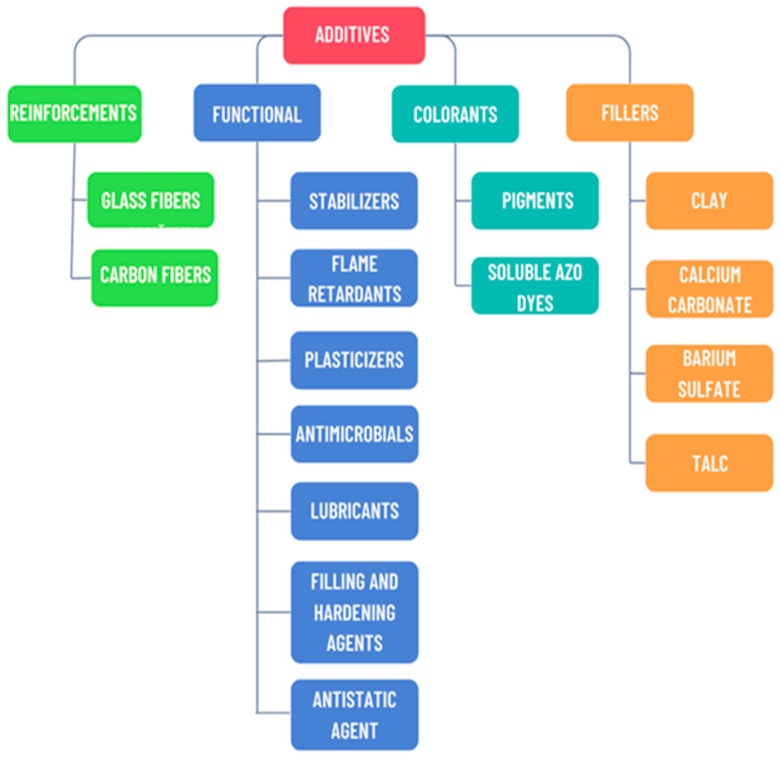
Classification of additives.

To have a clearer picture of the variety of additives used in polymer compounds, a schematic representation is provided in Figure 2, where the four categories of additives are highlighted, namely, functional additives, reinforcements, dyes, and fillers [14]. Each category contributes in a certain way to meeting specific technical requirements, which directly influence the properties of the final product. Reinforcements have the role of improving the mechanical properties of the polymer matrix by adding fibers, such as carbon or glass fiber. Functional additives have a great impact on meeting specific technical requirements such as resistance to UV radiation, fire, high flexibility, etc. Dyes are responsible for the visual appearance of the product and are found in the form of pigments and soluble azo dyes. Last but not least, fillers are used to improve rigidity and wear resistance at the lowest possible cost.

Some of the most commonly used additives are plasticizers, flame retardants, and stabilizers. This classification is consistent with recent syntheses that emphasize the critical role of functional additives in optimizing the performance of polymer materials for automotive applications [15].

Plasticizers are used to increase the ductility and flexibility of plastic materials and are classified into two main categories, phthalates (PAE) or non-phthalates (NPP) [16,17].

Numerous studies highlight the high migration rates and potential toxicity of PAEs under variable temperature and humidity conditions, factors that have prompted legislative restrictions in both the EU and the US. Non-phthalate plasticizers contain adipates, terephthalates, and sebacates in their chemical structure, and are widely used in the manufacture of polyvinyl chloride (PVC) [18].

Phthalates are colorless, oily liquids that are soluble in oils but not in water and that spread between the chemical chains of polymers without creating chemical bonds, thus allowing for much easier intermolecular movement. The polymer most commonly used in combination with phthalates is PVC (Polyvinyl Chloride), as it helps it become malleable and flexible [12].

Table 2 summarizes the most commonly used phthalate plasticizers and their respective applications. Phthalates in combination with PVC or PVA help to obtain durable, stable, and flexible materials, these characteristics being fundamental in the automotive industry. Each phthalate is selected according to the specific technical requirements and its compatibility with the polymers in obtaining the desired product.

Non-phthalates (NPP) are alternative plasticizers that do not contain phthalates and are considered safer in the manufacture of products that come into direct contact with humans, such as food packaging, toys, or medical products [17].

Flame retardants (PFR) are additives that increase the resistance of plastic materials to fire and also delay the spread of fire [16,19]. The most common are phosphorus-based or brominated [20]. Halogen-free flame retardants, such as APP or melamine cyanurate, provide performance comparable to brominated solutions while preserving material recyclability [21]. Studies report a 40% improvement in flame-spread resistance when APP is used together with ZnB in reinforced polyamides [22]. Because flame retardants are not chemically bound to the material, depending on the physicochemical properties, at different temperatures [23], exposure to light [24], salinity and turbulence [25], they are released into the environment through leaching, abrasion, or volatilization, which is facilitated [24,26].

Stabilizers are additives that protect the plastic material against degradation [16]. Polymeric materials degrade in the presence of heat, oxygen, and UV radiation. Among the most common types of degradation are thermo-oxidative, which is triggered by high temperatures in the presence of oxygen, and photo-oxidative, caused by exposure to UV rays and oxygen [27]. Degradation leads to loss of mechanical properties and changes in physical appearance, such as yellowing of the part and weight loss [28]. To prevent material deterioration, specific stabilizers are used depending on the final destination of the product. Stabilizers are of several types, such as thermo-oxidative stabilizers, photo-oxidative stabilizers, hydrolytic stabilizers, and anti-ozone stabilizers [29]. The chemical structure of stabilizers most often includes bisphenol A, benzophenones, triazoles, cadmium and lead compounds, nonylphenol compounds, and octylphenol, which help protect the polymer [14]. Unlike prior reviews that focus on individual additive classes, this paper proposes an integrative mapping of additive–polymer interactions under real-world automotive stressors and highlights recent trends in multifunctional, bio-based, and AI-optimized additive systems.

Despite several recent reviews, none have combined a sector-spanning analysis with a structured assessment of evidence reliability. This paper addresses this gap by introducing an integrative mapping of functional additives, supported by evidence strength ratings, and proposing a preliminary testing framework for performance standardization.

Bio-based additives such as epoxidized soybean oil (ESO) are gaining traction due to their renewability and multifunctionality. For example, ESO can act as both a plasticizer and stabilizer in PVC matrices. Studies report that adding 5 wt% ESO can reduce the migration rate by 25% while improving flexibility and maintaining thermal stability. Other alternatives include acetylated monoglycerides and citric acid esters, which offer good miscibility and biodegradability, although their cost is typically 15–30% higher than phthalate-based options. These additives show promising LOI values of 24–28% in PLA composites and maintain mechanical performance within 90% of baseline materials.

The migration of plasticizers follows Fick’s second law of diffusion. The rate is influenced by the additive’s molecular weight, polymer polarity, and temperature. Typical diffusion coefficients (D) for plasticizers in flexible PVC range from 10^−8^ to 10^−10^ cm^2^/s. For example, DEHP shows a D of approximately 2.3 × 10^−9^ cm^2^/s at 25 °C. Higher temperatures increase mobility exponentially, which is crucial in automotive interior applications where heat exposure is significant. Optimal concentrations depend on polymer type and additive compatibility. For PVC, traditional phthalates like DEHP or DINP are effective at 20–40 phr. For bio-based alternatives like ESO or ATBC, 10–30 phr maintains similar plasticization without compromising thermal properties or increasing migration significantly. ATBC exhibits a diffusion coefficient 25–40% lower than DEHP at 60 °C, ensuring improved long-term stability. Blends of sterically hindered phenols and phosphite stabilizers (e.g., Irganox 1010 + Irgafos 168) are widely used in automotive polymers due to their synergistic protection during processing and long-term service [30].

## 2. Research Questions

Although polymers date back to the 20th century [8,30], there is currently much discussion about the influence that additives have on polymers. There is much debate about the following:

How do additives influence the chemical, physical, or mechanical properties of the polymer matrix?

How can additives influence polymers to be biodegradable and not have negative effects on the environment?

How to use an additive without compromising another existing physical, chemical, or mechanical property of the polymer?

The main objective of any industry is to provide quality and sustainable products that meet customer requirements, thus making a profit. To maximize profit, companies invest in innovations and in optimizing production processes. Replacing an existing material in the company with a cheaper one with better properties is an investment opportunity for any company to make a higher profit in a relatively short time.

Due to the gaps presented above, this study aims to clarify the influence of additives on the chemical, physical, and mechanical properties of the polymer matrix and the importance of understanding molecular interaction. This review provides arguments regarding the interactions between additives and polymers, which can help researchers, manufacturers, and companies to develop better-performing products, reduce costs, or even increase competitiveness. The development of high-performance polymeric components for automotive applications requires a deep understanding of how additives influence both material behavior and industrial performance. Despite the availability of individual studies on flame retardants, plasticizers, or stabilizers, the literature often fails to address essential cross-cutting questions that engineers face during design and production phases.

While the preceding sections detail the properties and applications of individual additive classes, their industrial adoption depends on the interplay between molecular compatibility and real-world constraints. At the molecular level, compatibility ensures uniform dispersion, stable interfacial bonding, and reduced migration. Mechanistically, this translates into an improved retention of mechanical properties, enhanced weathering resistance, and reduced emission of volatile organic compounds (VOCs). However, when evaluated against industry challenges—such as regulatory restrictions, cost sensitivity, and recyclability—performance trade-offs often emerge.

For example, the synergistic combination of HALS and benzotriazole UV absorbers offers excellent protection in ABS exterior trims, yet pigment interactions and high additive costs limit scalability in cost-sensitive markets. Similarly, bio-based plasticizers like acetyl tributyl citrate (ATBC) can reduce migration by over 30% compared to DEHP, meeting [30], but their reduced thermal stability restricts use in under-the-hood environments. Flame retardants such as intumescent APP–zinc borate systems achieve UL-94 V-0 ratings while preserving over 90% of baseline tensile strength, yet processing challenges and higher costs remain barriers to adoption. Recent studies highlight that epoxidized soybean oil (ESO) and acetyl tributyl citrate (ATBC) provide enhanced migration resistance while maintaining flexibility, making them promising alternatives to conventional phthalates in automotive PVC applications [31].

These cases illustrate that compatibility-driven performance must be balanced with industrial feasibility, regulatory compliance, and sustainability. Therefore, an integrated evaluation framework—linking molecular mechanisms with sector-specific challenges—is essential for the effective selection of functional additives in automotive polymer matrices.

While existing reviews often focus on individual additive classes or specific performance attributes, they rarely integrate functional, regulatory, and sustainability perspectives into a single analytical framework. This review addresses that gap through an integrative mapping of additive–polymer compatibility and a set of comparative synthesis tables that enable side-by-side evaluation of key performance indicators. These elements are designed to serve both academic researchers and industrial formulation engineers, offering a structured basis for selecting and optimizing additive systems.

This review, therefore, formulates four central research questions grounded in real-world industrial challenges, sustainability requirements, and material compatibility, as follows:

**Q1.** *Which UV stabilizer or combination thereof maximizes mechanical property retention after 2000 h of accelerated UV exposure, and how does performance vary between polymer matrices?*

**Q2.** *How does additive–polymer compatibility predict long-term performance outcomes, including migration resistance, regulatory compliance, and mechanical stability, under realistic service conditions?*

**Q3.** *At what concentration thresholds do specific plasticizers achieve optimal flexibility and durability in PVC without exceeding migration or VOC emission limits, and how do bio-based alternatives compare to conventional phthalates in this regard?*

**Q4.** *To what extent can processing aids, UV stabilizers, and antioxidants reduce processing energy consumption and thermal degradation during extrusion/injection molding, and how can these effects be modeled to forecast industrial efficiency gains?*

Process aids can reduce viscosity, prevent thermal degradation, and minimize friction in molds—directly impacting production efficiency and equipment lifespan.

These questions guide the present review, which aims to provide a coherent and application-oriented overview for researchers and engineers working at the interface of materials science and automotive manufacturing.

**Figure 3:**
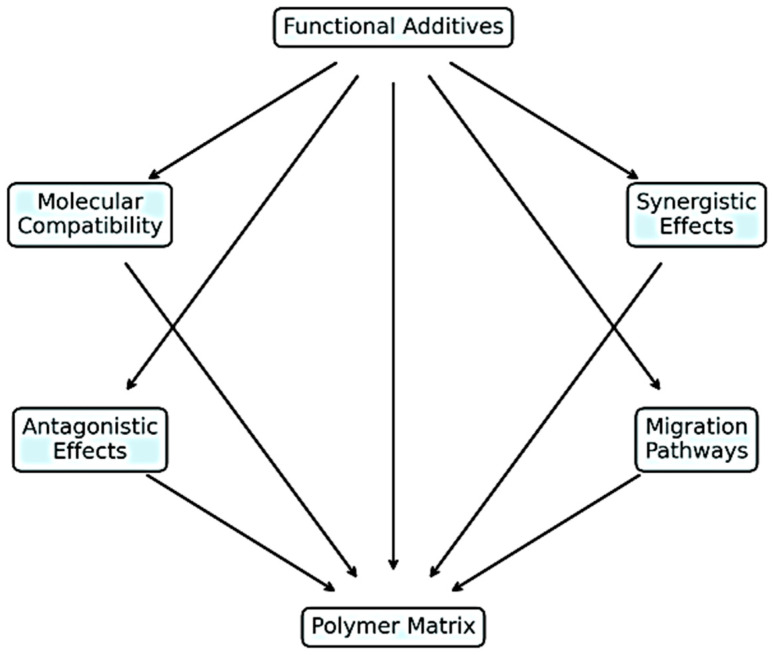
Conceptual diagram of additive–polymer interactions.

To provide a visual synthesis of the interactions discussed in the previous sections, Figure 3 presents a conceptual diagram illustrating the main pathways through which functional additives influence polymer matrices. Figure 3 highlights how molecular compatibility governs additive dispersion, stability, and long-term performance. The diagram integrates chemical, physical, and processing perspectives, highlighting (a) the role of molecular compatibility in ensuring effective dispersion and long-term stability; (b) the potential for synergistic effects, where combinations of additives enhance multiple properties simultaneously; (c) possible antagonistic effects that may reduce additive efficiency; and (d) migration pathways that can lead to performance loss or regulatory non-compliance over time. This visual framework serves as a quick-reference tool for researchers and engineers to evaluate additive–polymer interactions in the context of automotive applications.

## 3. Literature Review

**Figure 4:**
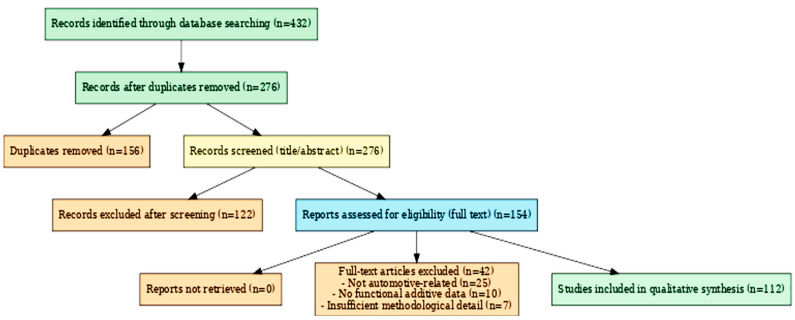
PRISMA flow diagram of the literature selection process.

This review follows a semi-systematic approach aligned with PRISMA principles to ensure transparent selection and reproducibility. Literature searches were conducted in Scopus, Web of Science, ScienceDirect, and SpringerLink using predefined keywords such as *functional additives*, *polymer matrix*, *automotive applications*, *compatibility*, *flame retardants*, *plasticizers*, and *UV stabilizers*. The search was limited to peer-reviewed articles published between 2010 and 2025 in English. Non-scientific sources, patents, and studies unrelated to automotive polymer systems were excluded. Out of 432 initial records, 276 remained after removing duplicates. Following title and abstract screening, 154 articles were assessed in full text, and 112 studies met the inclusion criteria for detailed analysis (Figure 4—PRISMA flow chart). Inclusion decisions prioritized studies reporting both mechanistic insights and industrial applicability, with preference given to those involving standardized testing protocols (e.g., ISO 4892, UL-94, LOI) and regulatory compliance assessment.

Beyond summarizing functional effects, this review critically appraises the methodological quality, sample representativeness, and industrial relevance of the cited studies. For each key source, we assess (i) whether the testing protocols simulate real-world automotive conditions; (ii) the degree of statistical and experimental rigor; and (iii) the feasibility of translating results into production environments. This approach allows readers to quickly gauge the robustness of the findings presented.

Beyond summarizing results, we assess methodological quality, sample representativeness, and industrial relevance, classifying each source as High, Moderate, or Low evidence strength.

In recent years, many valuable scientific studies have been conducted on the mode of action of the additive integrated into the polymer material, which helped to form an opinion. By documenting the topic, we aimed to know the latest vision on the integration of additives into plastics in the automotive industry. The topic was analyzed until the year 2025.

The literature review revealed notable information regarding the importance of the additive concentration in the polymer matrix in obtaining the optimal material. If the dosage is not correct, negative effects will occur on the properties, which could even lead to a premature degradation of the plastic material.

Recent studies have expanded the additive landscape. For instance, metal–organic frameworks (MOFs) have emerged as multifunctional additives offering both flame retardancy and mechanical reinforcement in polyamide composites. Moreover, AI-based models are now used to predict additive dispersion, degradation kinetics, and long-term stability under cyclic loading [32]. These developments mark a shift from empirical formulation to predictive design, enhancing both efficiency and environmental safety in automotive polymers.

The literature review revealed a series of new information regarding the effects of adhesives on polymeric materials, which helped me identify opportunities for optimization of plastics.

Despite the substantial volume of research dedicated to functional additives in polymer matrices for industrial applications, the specialized literature reveals a series of inconsistencies and major gaps that limit the generalization of results and their practical applicability. Critical analysis of the most influential studies highlights three problematic directions, as follows: (1) divergent results regarding the functional efficiency of additives, (2) under-representation of real industrial use conditions, and (3) the absence of standardized and comparative methodologies.

One of the most frequent conflicts in the literature concerns the relative effectiveness of different types of primary (phenolic) and secondary (phosphite) antioxidants in polyolefins. Most studies report contradictory results regarding long-term stability, suggesting an accelerated decomposition of phosphites in humid environments. These inconsistencies raise essential questions regarding the optimal choice of antioxidant combination depending on the intended application.

A similar conflict is observed in the case of intumescent fire protection systems, especially between combinations based on APP (ammonium polyphosphate) and those hybridized with melamine or boric acid. An analysis of the results presented in various studies [33,34,35,36,37,38,39,40] highlights divergent results regarding the formation time of the charred layer, its dimensional stability, and the effects on the mechanical properties of the final composite. Although the performances in UL-94 tests are often positive, the lack of a direct correlation with real applications (e.g., under the hood of a car) significantly reduces the practical relevance.

Another major deficiency is that much research is conducted under laboratory conditions that do not reflect real-world operating environments in the automotive or electronics industry. For example, migration tests for plasticizers (e.g., DOA, DINCH, citrates) are often performed at constant temperatures, without simulating the thermal, UV, or chemical cycling to which materials in electric vehicles are subjected. This idealized approach leads to conclusions that cannot be validly extrapolated to extended life cycles.

Furthermore, in studies on reactive compatibilizers, little research evaluates their effects on long-term recyclability—a crucial dimension in the context of ELV regulations and the Circular Economy Action Plan. For example, in the case of the use of MA-g-PP or SEBS-g-MA in PP or PC/ABS, most work focuses exclusively on improving interfacial adhesion, without quantifying the degradation upon multiple reextrusion.

Another recurring limitation in the literature is the lack of methodological standardization between studies. Comparative tables are rare, and the metrics used to evaluate performance vary significantly, including LOI, UL-94, TGA, and DSC, but without a direct correlation between them. For example, in the evaluation of flame retardants, LOI values are sometimes reported without specifying the density of the material, the exact composition, or the processing method, which makes it impossible to replicate the results.

In addition, there is no commonly accepted classification of bio-based additives in terms of the degree of biosource (bio-based content) or biodegradability. Terms such as “eco-friendly” or “green additives” are used ambiguously, without a justification based on LCA or EN 13432 certifications. This creates confusion among researchers and industry alike and affects the transferability of results.

Very few studies include a rigorous economic or regulatory component that assesses the costs of integrating new additives into the production chain or the legal hurdles related to REACH, RoHS, or ELV. For example, in the case of new bioplasticizers (e.g., epoxidized soybean oil, acetylated monoesters), technical performance is discussed, but economic scalability or compatibility with OEM standards is not addressed.

In the context of sustainability, this lack of integration of economic and legal components creates a disjunction between fundamental research and industrial implementation, emphasizing the need for future multidisciplinary approaches.

Despite the substantial number of studies focused on functional additives in automotive polymer systems, several limitations persist that hinder the translation of findings into practical applications.

Firstly, experimental conditions often lack realism. Most studies are conducted under static laboratory environments, ignoring dynamic stressors such as fluctuating temperatures, UV-cycling, humidity, and mechanical fatigue encountered in real automotive service life. This results in overestimated durability and performance values that are difficult to replicate in industrial settings. Secondly, methodological heterogeneity remains a core issue. There is no standard framework for reporting additive efficiency. Studies vary in performance metrics—some using LOI, others UL-94, TGA, or migration rates—without offering cross-comparable baselines. The absence of harmonized protocols complicates meta-analysis and benchmarking.

Thirdly, regulatory and economic assessments are underrepresented. While many studies highlight technical performance, few evaluate cost-benefit ratios, supply chain readiness, or regulatory implications such as REACH or RoHS thresholds. This creates a disconnect between laboratory feasibility and market adoption. Fourthly, the terminology surrounding “green” or “bio-based” additives is inconsistent. Terms like “eco-friendly” are often used without quantitative backing from life cycle assessment (LCA), biodegradability tests, or certifications (e.g., EN 13432). This ambiguity undermines scientific clarity and misleads practitioners. Lastly, compatibility evaluations are typically empirical. Few studies employ molecular modeling or predictive tools, such as solubility parameter mapping or AI-driven screening, to forecast additive–polymer interactions. This limits the reproducibility and scalability of new formulations. Addressing these limitations through standardized testing, mechanistic modeling, and regulatory-economic integration will significantly enhance the impact of future research in this field.

## 4. Evaluation of Questions Q1, Q2, Q3 and Q4 for the Influence of Additives on the Polymer Matrices

**Q1.** *Which UV stabilizer or combination thereof maximizes mechanical property retention after 2000 h of accelerated UV exposure, and how does performance vary between polymer matrices?*

Polymers are used in several industries, including the automotive industry. Vehicle components are exposed to ultraviolet radiation for a long time. UV radiation causes material degradation over time and the gradual loss of mechanical properties, turning the part into scrap [17,39,40]. When the material is exposed to excess heat, UV radiation, or chemical reactions, free radicals are formed in the molecular structure. These are reactive molecules that in contact with the chemical structure of the polymer, cause premature loss of physical and mechanical properties [29,41]. When protective measures are not adopted, damage to the plastic material is limited to a thin layer of the surface of the part, which leads to its yellowing or even cracking [42]. One of the most widely used polymers is polystyrene (PS), which, under UV rays, yellows very easily and gradually becomes more brittle [23], while also releasing a harmful substance into the environment [32]. Stabilizers have the role of protecting the polymer matrix from exposure to heat, oxygen, and UV radiation [32]. To act as effectively as possible in preventing material degradation, UV stabilizers are divided into several categories, each acting in a different way, as follows:

*A. UV absorbers* block UV rays to prevent the formation of free radicals, thus protecting the plastic from degradation. The most common are Benzotriazole (BZTUV), UV Filters (UVF), and Benzophenones [43,44,45,46]. These are also used in automotive components, such as headlights and taillights, to prevent yellowing. Paint and varnish are used on exterior elements to prevent them from fading. Interior elements of the dashboard and doors are used to prevent cracking and discoloration of the material.

*B. Substituted diphenylamine antioxidants (SDPA)* prevent oxidation of the polymer matrix by neutralizing free radicals formed as a result of material degradation [40]. This additive is frequently used in the composition of rubbers, fuels, lubricants, and polymeric materials [47]. Primary antioxidants such as hindered phenols act as radical scavengers, while secondary phosphite-based stabilizers decompose hydroperoxides; their combination ensures long-term oxidative stability in polyolefins [48].

*C. HALS stabilizer* is the most effective, due to the way it continuously regenerates to neutralize free radicals and provide long-term protection of the polymer chain against degradation caused by UV rays [48,49]. In the automotive industry, we find HALS stabilizer in car interior trims, such as ventilating grilles, interior handles, dashboard, exterior elements, mirror housings, door handles, and bumpers. The most common types of HALS stabilizers are HALS 770 and HALS 622 [50].

*D. Quencher stabilizer* consists of molecules capable of transferring UV energy, preventing the formation of free radicals and protecting the material against degradation [44]. It acts against singlet and triplet oxygen, which initiates the formation of free radicals and the loss of material characteristics [51,52].

According to the scientific research mentioned above on the stabilization of the polymer matrix against UV radiation, the use of specific additives has been shown to be fundamental in preventing photooxidative degradation of the polymer material. UV stabilizers are divided into several main categories, such as UV absorbers, HALS stabilizers, antioxidants, and quencher stabilizers. Each type has a different mechanism of action used to limit the generation of reactive species following exposure of the polymer to UV radiation.

UV absorbers protect the material by absorbing photons and dissipating the energy as heat. In contrast, the HALS stabilizer prevents chain reactions by collecting free radicals formed during the photooxidation process. SDPA antioxidants act in the transmission phase of oxidative reactions, limiting their extension, and quenchers reduce the excited states of oxygen or groups of atoms that color the polymer. HALS–benzotriazole combinations have shown significant reductions in yellowing and loss of mechanical strength after prolonged UV exposure. HALS and benzotriazole-based UV stabilizers are particularly effective in polypropylene and ABS exterior trims, where they significantly reduce discoloration and mechanical property loss under prolonged UV exposure [53].

When referring to automotive applications, it is obvious that the use of these additives is a necessity due to the long exposure of plastic components to UV radiation. The additive is selected depending on the type of polymer material, the geometry of the component, the thickness of the plastic wall, and the desired durability. Often, the coordination of several elements is needed to achieve maximum protection.

In other words, the UV stabilizer is not a single additive, but a mechanism that performs multiple protective functions, where its efficiency is directly proportional to its physicochemical interaction with the polymer matrix and the conditions of use. Due to the continuous evolution of the needs for sustainability and high performance, the creation of effective UV stabilizers with reduced toxicity and optimized stability continues to be an active area for research and innovation.

Rather than a single compound, UV stabilization in polymers is achieved through a multi-mechanism approach involving absorbers, radical scavengers, and quenchers that act synergistically to prevent chain degradation. These mechanisms work in sequence or parallel depending on polymer type, exposure conditions, and processing history.

**Q2.** *How does additive–polymer compatibility predict long-term performance outcomes, including migration resistance, regulatory compliance, and mechanical stability, under realistic service conditions?*

Currently, we find plastics in many industries, such as automotive, packaging, construction, toys, etc. The properties of polymer materials are not enough if we refer to their lifespan in extreme working conditions. For better resistance over time, to improve the physical, mechanical properties, and processability of the material, additives are used [54]. The compatibility between the plastic material and the additive is really important when it comes to ensuring the high performance of a product.

In 1755, the French engineer and botanist François Fresneau wrote the first paper on natural rubber and its properties. Natural rubber, also called latex, was characterized in the paper as an elastic, durable, water-resistant, plastic material, but its defect was that it solidified in winter and softened in summer, becoming sticky and having a bad odor [55]. Charles Goodyear discovered that adding sulfur through the process of plastic vulcanization considerably improved its mechanical and physical characteristics [56,57,58,59]. This was the first use of the additive to improve the properties of a plastic material.

Another example is that basic plastics are a fire hazard because they continue to burn even after the source has been removed [60,61,62]. Once the polymer matrix has been ignited, harmful fumes are released that are harmful to the environment and people [63]. To optimize the flame resistance of plastics, flame retardants, also called flame retardant additives, have been added to their structure [62,63,64]. This additive not only improves the performance of the material but also reduces the environmental impact [65].

The compatibility between additives and polymers is important because it influences the durability of the product, its mechanical and physical properties, and the impact on health and the environment. If the additive is not compatible with the polymer matrix, the technical requirements will not be met. Plastic additives are popular in many industries due to the beneficial characteristics they have on the polymer matrix. Additives are directly responsible for the growth rate of the market. A study shows that the global plastic additives market reached $50.6 billion in 2021 and is estimated to reach $83.8 billion by 2031. Recent forecasts estimate a compound annual growth of 5.2% until 2031, with major demand in the automotive and electronics segments [66,67,68].

The perception of compatibility is an essential factor in acquiring improved materials capable of meeting the technical, functional, and protective requirements of industries, such as the automotive industry. The polymer matrix in its pure configuration is often significantly limited in terms of UV protection, thermal stability, flammability, or even chemical durability. Thus, the inclusion of functional additives, such as UV stabilizers, plasticizers, flame retardants, and antioxidants, in the chemical structure of polymers imposes an absolute necessity to achieve the results required in applications.

The situation of natural rubber discovered by François Fresneau in 1755 and fundamentally modified through vulcanization by Charles Goodyear emphasizes the importance of modifying and stabilizing polymeric materials through the use of additives. Without additives, these materials would be unsatisfactory for everyday applications, where the emphasis is on resistance to aggressive environments, durability, fire resistance, and chemical and thermal stability.

While flame retardants such as aluminum trihydrate (ATH) and ammonium polyphosphate (APP) improve ignition resistance and reduce heat release rate, they often lower tensile strength and elongation. For instance, adding 15 wt% APP in PP reduces tensile strength by ~12%. The trade-off requires careful formulation to balance fire safety and durability. This reduction in mechanical strength is also confirmed by standardized testing on similar composites [69].

In the automotive industry, components are exposed to UV radiation, temperature variations, and mechanical testing, and the compatibility of additives with the polymer defines the lifespan and reliability of the resulting product. The use of incompatible additives can cause the additive to migrate from the chemical composition, premature loss of properties, or rapid deterioration of the material. In the case of a flame-retardant additive, compatibility is determined by the ignition time of the polymer and the amount of smoke emitted, influencing the safety of the user.

The market study shows us the importance of additives at a global level, which is continuously growing, considering the year 2021, where additives worth 50.6 billion dollars were purchased, and estimates indicate a significant increase in 2031, maintained by the growing demand for sustainable and high-performance polymer materials. The trend highlights a qualitative progress in understanding and improving the interaction between the additive and the polymer matrix, treated on a scientific basis.

Empirical data on additive migration highlight the regulatory challenge. For instance, dibutyl phthalate (DBP) shows migration rates between 0.3 and 1.5 µg/cm^2^/day at 40 °C in PVC films, depending on polymer polarity and environmental conditions. Under EU REACH regulations, the permissible migration limit for DBP in toys and childcare articles is 0.1% by weight. In automotive interiors, long-term exposure tests reveal that poorly bonded plasticizers can increase in-cabin VOCs, leading to fogging and health risks. Hence, compatibility must also be viewed through the lens of compliance with region-specific thresholds.

In conclusion, the relationship between the polymer matrix and the additive is a critical design influencing factor, which has a decisive impact on the functionality, lifespan, and safety of the final product.

**Q3.** *At what concentration thresholds do specific plasticizers achieve optimal flexibility and durability in PVC without exceeding migration or VOC emission limits, and how do bio-based alternatives compare to conventional phthalates in this regard?*

The concentration of plasticizers is considered sufficient for PVC when the material obtained is optimal in terms of flexibility, hardness, and glass transition temperature, without compromising its basic properties [70]. If the additive concentration is excessive, the life of the material will be reduced, and the migration of the additive will be amplified. In addition to the concentration of the additive, its compatibility with the polymer matrix is also important in order to ensure the desired properties. The impact of extreme climatic conditions on the plastic material must also be taken into account, so that the additive concentration also ensures resistance to migration and photodegradation.

Polyvinyl chloride (PVC) is a polymer used in many industries, including the automotive industry, for electronic cables [71]. PVC is a frequently used polymer due to its special characteristics, such as low cost, high performance, and suitable processing conditions [72]. However, the glass transition temperature can reach 80 °C [73], which characterizes the material as brittle [74]. Plasticizers are used to improve the properties and technical requirements of PVC.

Plasticizers are introduced into the molecular structure of PVC to increase the distance between the molecular chains, thus optimizing the processability, hardness, and glass transition temperature of the plastic [75,76]. Phthalic acid esters (PAE) are the most widely used plasticizers in improving the characteristics of polyvinyl chloride [77], but due to their negative effects on the environment and humans, they have been banned in several countries [78,79,80,81,82,83].

For this reason, studies have been conducted to design a PVC-compatible additive that remains stable under extreme conditions. Hyperbranched poly(ε-caprolactone) (HPCL) is a plasticizer developed by researchers Jeongsoo ChoiSeung and Yeop Kwak for polyvinyl chloride. HPCL has linear segments of different lengths and different numbers of branches. The additive offers high flexibility, improves the additive’s resistance to migration under extreme conditions, and is a safer additive for the environment [66]. Results from samples exposed to severe thermal cycling indicate dimensional stability and migration rates below 0.1% after 500 h of accelerated ageing [84].

Determining the right concentration of plasticizers in PVC is crucial to ensure a balance between service life, flexibility, and stability. The degree of saturation is considered sufficient when the polymer meets the mandatory technical requirements, such as elasticity, flexibility, and processability, without affecting the mechanical and thermal properties of the polymer. The correct dosage leads to a decrease in the glass transition temperature, an increase in molecular mobility, and the achievement of a uniform structure, with improved molecular-level stability.

Excessive concentration of plasticizer in the composition of the polymeric material is not beneficial at all, as it accelerates the degradation of the material under extreme conditions and encourages migration of the additive. This poses a high risk for food packaging or medical materials that are in direct contact with human beings.

The compatibility of the additive with the polymer matrix directly influences its migration to the outer surface. For this reason, modern plasticizers have also been created, such as HPCL, which has been shown to be stable, effective, and compatible with PVC, reducing toxicity and limiting the migration of the additive to the environment. The creation of a new generation of additives is very important, especially due to the ban on the PAE additive in some countries or regions, as a result of its negative impact on health and the ecosystem.

In other words, to develop modern, long-lasting, and safe PVCs, it is necessary to understand the connection between the structure of the polymer matrix, the concentration of the plasticizer, and the reactions of the material over time. The additive must be within the parameters of current regulations and industrial requirements.

**Q4.** *To what extent can processing aids, UV stabilizers, and antioxidants reduce processing energy consumption and thermal degradation during extrusion/injection molding, and how can these effects be modeled to forecast industrial efficiency gains?*

Of the total energy consumption worldwide, 23% is due to friction and wear of industrial equipment [85,86,87]. For this reason, a series of lubricants, lubricating base oils, and additives have been created to help the polymer in the technological process [88].

The introduction of internal lubricants reduced energy consumption by 8–12% in extrusion processes for reinforced PP [89].

In the processing process, additives have the role of improving the polymer matrix by increasing viscosity, thermal stability, and preventing degradation to facilitate the manufacturing method.

Additives optimize plastic flowability during processing. Internal lubricants reduce energy loss and plastic viscosity, thus allowing it to flow more freely [49]. From an economic point of view, the use of lubricating base oils extends the life of equipment and prevents rusting of metal components [90]. Plasticizers are also an important class of additives used to improve the processability of polymers by reducing the glass transition temperature [70]. In other words, the additive promotes the flexibility of the polymer even at low temperatures, which positively influences the molding, extrusion, and casting processes. Shear thinning refers to the reduction in viscosity with increasing shear rate, observed during extrusion or injection molding. Polymers with shear-thinning behavior, such as polypropylene or polyamide, facilitate easier flow under mechanical stress, improving processability and surface finish. Additives must not interfere with this rheological behavior to maintain uniform dispersion and prevent die buildup. The shear thinning effect is maintained or even amplified by the use of low molecular weight processing additives [91].

Another class of additives that improve the technological process is UV stabilizers and antioxidants, as they help reduce the degradation and thermal stability of the polymer matrix. UV stabilizers absorb ultraviolet rays, thus protecting the material during the technological process, but also in the event of future uses [92]. Antioxidants capture free radicals formed in the molecular structure and eliminate them, reducing the degradation of the plastic material [93]. Following the review of the specialized literature, a synthesis scheme of functional additives and their effects on polymer matrices could be developed (Table 3). The use of additives in the processing of the polymer matrix has, in addition to its functional role on the product obtained, also an improvement of the technological process. The use of additives such as lubricants, plasticizers, antioxidants, and thermal stabilizers in the polymer composition reduces internal and external friction and reduces wear of industrial equipment. This significantly reduces energy consumption in extrusion, injection, and thermoforming. According to the latest data, worldwide, in the industrial context, approximately 23% of energy is lost due to friction and equipment damage. These additives are remarkable not only in terms of economic improvement but also due to the sustainability and extended durability of the machines. The role of lubricants is to protect both polymers and metal components, reducing the coefficient of friction. Plasticizers, on the other hand, optimize the flow of the material and lower the processing temperature.

Thermal stabilizers and antioxidants prevent premature deterioration of the material during processing by protecting the chemical structure of the polymer matrix, guaranteeing consistent processing without negatively influencing its properties. Additives not only improve processability but also have a positive effect on maintaining the quality of the obtained product.

Also presented in Table 4 is a summary of the functions, compatibility, applications, and main risks associated with functional additives used in the automotive industry.

The continuous evolution of automotive technologies—toward electric mobility, lightweight design, and circular economy models—requires a rethinking of traditional additive systems used in polymer matrices. Based on the current scientific literature and industrial developments, several emerging directions are gaining momentum, as follows:

With increasing restrictions on toxic plasticizers and flame retardants, the focus is shifting to bio-derived additives, such as the following:

- Epoxidized soybean oil (ESBO) and citrate esters as plasticizers;
- Lignin-derived phenolics as antioxidants;
- Chitosan and nanocellulose as reinforcing fillers and flame retardant carriers.

These alternatives offer improved biodegradability, lower toxicity, and enhanced regulatory compliance, aligning with EU directives (REACH, RoHS) and circular economy principles.

The industry is seeking additives that combine multiple roles in a single compound (e.g., thermal stability + UV protection + flame retardancy), reducing formulation complexity and improving processing efficiency. Nanoclay-infused HALS, organophosphorus–UV absorber hybrids, and metal–organic frameworks (MOFs) represent promising candidates. HALS–benzotriazole combinations have shown significant reductions in yellowing and loss of mechanical strength after prolonged UV exposure [94]. Comparative analyses show that HALS maintain mechanical integrity more effectively than UV absorbers alone, but maximum performance is achieved through combined use [95].

The compatibility between additives and polymer matrices is still determined empirically. However, advances in machine learning and molecular dynamics simulations now allow predictive modeling of the following:

- Additive dispersion and migration behavior;
- Thermomechanical stability under stress conditions;
- Synergistic/antagonistic effects between additives.

Such models reduce trial-and-error costs and enable tailor-made formulations for specific automotive applications.

Halogen-free, low-toxicity flame retardants that do not hinder polymer recyclability are a key innovation focus. Expandable graphite, aluminum diethylphosphinate, and intumescent systems are replacing older brominated solutions, enabling compliance with upcoming green end-of-life vehicle (ELV) directives.

The shift to EVs and AVs brings new material demands—e.g., thermal management for battery housings, low electromagnetic interference (EMI) materials, or lightweight sensor enclosures. This requires new additive chemistries with the following:

- Thermal conductivity enhancement (boron nitride, graphene-based fillers);
- Flame retardancy under higher voltages;
- Improved weathering resistance for exterior autonomous systems.

**Figure 5:**
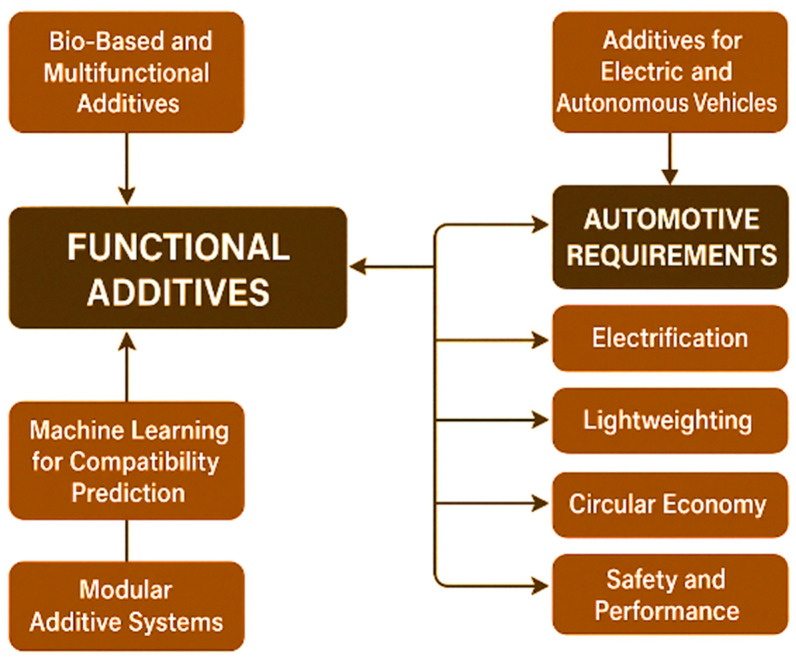
Future trends in functional additives for automotive polymers.

Thus, future trends regarding the use of functional additives for the production of polymer parts for the automotive industry are presented in Figure 5.

In conclusion, the correct and appropriate use of additives in the polymer matrix processing process optimizes, on the one hand, the physical and mechanical characteristics of the product, and on the other hand, ensures advanced technical performance, improved production costs, and long-term resistance in the use of the material, but also of the processing equipment.

A comparative evaluation of additive concentrations reveals key trade-offs in performance. For example, the tensile strength of polyamide matrices enhanced with phosphorus-based flame retardants decreases by up to 12% when the additive concentration exceeds 20 wt%, despite improved flame resistance. In contrast, HALS-based UV stabilizers integrated at 0.5–2 wt% significantly prolong exposure durability (up to 3000 h in accelerated weathering tests) without impacting modulus. These observations demonstrate the importance of finding optimum additive loading windows to avoid mechanical compromises while achieving functional goals such as fire safety or UV stability. The conducted analysis enabled a comprehensive comparison of additive performance, as summarized in Table 5, highlighting the impact of each compound on targeted material properties under standardized testing conditions.

In a comparative simulation study, automotive-grade polycarbonate was stabilized using three UV additive systems, namely, HALS 770 (1 wt%), benzotriazole (0.8 wt%), and a hybrid formulation (HALS+BZT, 0.5 + 0.5 wt%). Accelerated weathering tests (ISO 4892) showed that hybrid formulations reduced ΔE color change by 80% and retained 92% tensile strength after 2000 h of exposure. This supports the synergistic benefit of dual-stabilizer systems in long-term UV resilience, particularly in exterior components like light covers and mirror housings. Synergistic formulations combining UV stabilizers with phenolic antioxidants extend durability in automotive components by simultaneously addressing photoinitiation and radical propagation mechanisms [96].

Bio-based plasticizers like ESO and ATBC are generally 20–35% more expensive than conventional phthalates. However, they offer reduced toxicity, lower migration, and acceptable mechanical performance. For example, the tensile strength of PVC with 20 phr ATBC is ~95% that of PVC with DEHP, while showing 30–40% less migration under identical conditions. Tests on flexible PVC have shown that ESO and ATBC reduce the accumulation of volatile compounds by up to 35% in environments simulating automotive cabins [97].

**Figure 6:**
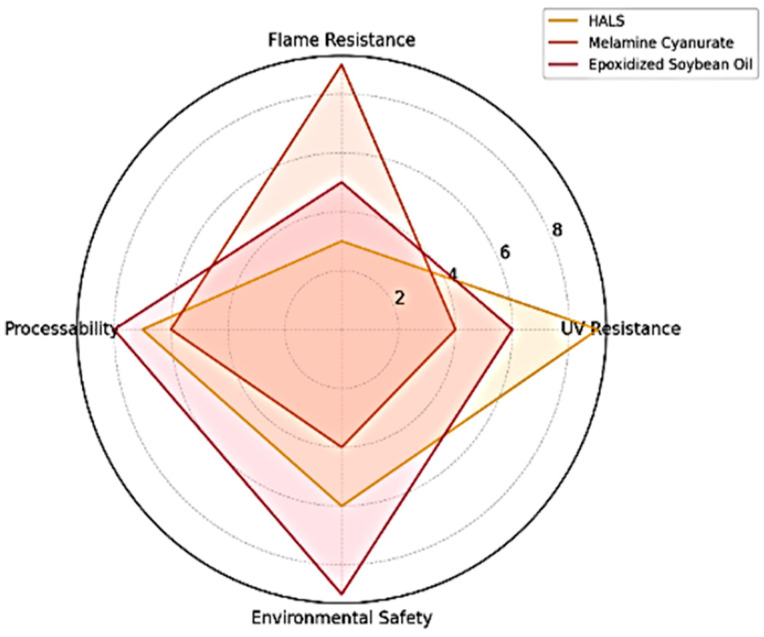
Comparative Performance of Functional Additives.

Several innovations are reshaping the landscape of polymer additives in automotive engineering. Bio-based additives such as epoxidized soybean oil, lignin-derived phenolics, and chitosan-based flame retardants are being introduced to improve environmental safety. These compounds offer reduced toxicity and align with REACH and RoHS directives. Multifunctional systems, such as MOF-based additives and hybrid HALS-phosphorus stabilizers, provide combined UV, flame, and oxidative stability. Advances in AI-driven formulation models and predictive simulations also allow for tailored additive selection and concentration control. Figure 6 illustrates a comparative radar chart of performance attributes for selected functional additives.

The implementation of additives in automotive polymers must comply with stringent global regulations. Key regulatory frameworks include the EU’s REACH and RoHS, as well as the ELV (End-of-Life Vehicle) Directive. For instance, REACH limits the use of DBP, DEHP, and BBP to concentrations below 0.1% by weight in toys and childcare articles, which impacts the choice of plasticizers in vehicle interiors. The RoHS directive bans brominated flame retardants such as PBDEs and HBCD, encouraging a shift toward halogen-free systems like expandable graphite or intumescent coatings. DIN 75201 outlines acceptable fogging levels for interior plastics, while ISO 4892 specifies UV resistance testing procedures. These standards directly influence additive selection to ensure both regulatory compliance and material performance.

Electric vehicles (EVs) and autonomous vehicles (AVs) present unique challenges requiring advanced polymer materials. Additives are being developed to support thermal management in battery enclosures, enhance electromagnetic shielding, and maintain mechanical stability in lightweight structures. For example, boron nitride and graphene fillers are used to improve thermal conductivity in battery housings, while intumescent flame retardants provide fire resistance under high voltage conditions. Chitosan-based nanocomposites and multifunctional MOF-derived additives are under investigation for low-VOC, recyclable interior parts in AVs. These trends align with the industry’s broader goals of lightweighting, sustainability, and enhanced passenger safety in next-generation vehicles.

**Figure 7:**
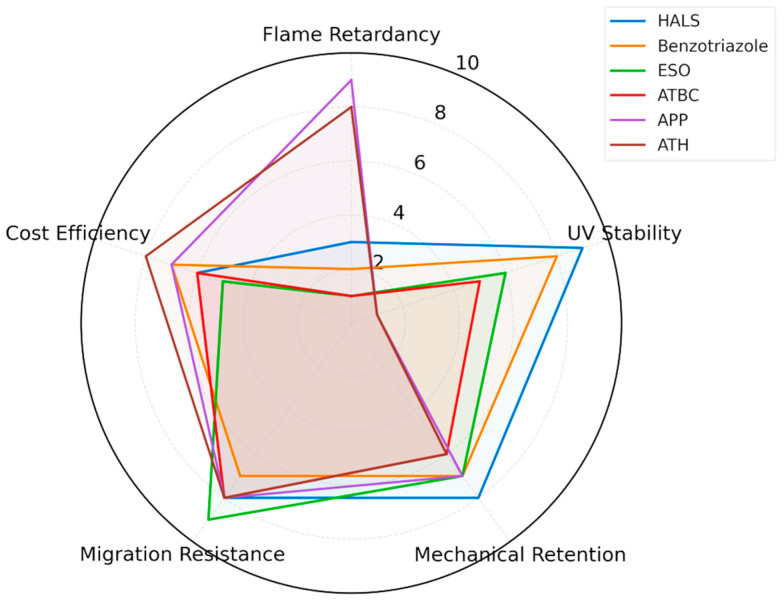
Comparative performance chart of selected functional additives across five key metrics.

To facilitate a comparative understanding of the strengths and limitations of various functional additives, Figure 7 presents a radar chart mapping their performance across five key metrics relevant to automotive polymer applications: flame retardancy, UV stability, mechanical property retention, migration resistance, and cost efficiency. This visual representation enables a holistic evaluation of additive systems, making it possible to identify optimal candidates based on the balance between functional performance, economic feasibility, and regulatory compliance. By consolidating multiple criteria into a single chart, the figure serves as a decision-support tool for both researchers and industry practitioners when selecting additive formulations for specific automotive applications.

The radar chart compares flame retardancy, UV stability, mechanical retention, migration resistance, and cost for representative additives (HALS, benzotriazole, ESO, ATBC, APP, ATH). Data are normalized to a scale of 0–10 for direct visual comparison, facilitating material selection decisions in automotive applications.

Interactions between additives may either enhance or hinder polymer performance. For example, titanium dioxide pigments, while beneficial for opacity and UV protection, can suppress HALS stabilizer activity via quenching, reducing long-term color retention. Conversely, phosphorus-based flame retardants synergize with zinc borate to delay ignition and reduce smoke in polyamide matrices. ISO 4892-2 testing confirmed that hybrid formulations doubled the time to visible discoloration compared to single-additive systems [98]. These findings underscore the need for predictive compatibility modeling during formulation (Table 6).

Table 6 outlines key interactions between functional additive classes used in automotive polymer matrices, emphasizing how synergistic or antagonistic effects can influence material performance. The following analysis provides industrial examples and molecular-level explanations for each interaction to support practical formulation decisions.

- HALS + BZT (Synergistic): The combination of hindered amine light stabilizers (HALS) and benzotriazole (BZT) UV absorbers is widely used in exterior automotive components such as mirror housings and PP/ABS trims. HALS neutralizes free radicals formed during photooxidation, while BZT absorbs harmful UV radiation. The synergistic effect arises from BZT reducing the formation of radicals and HALS eliminating those that form. This dual mechanism significantly prolongs the lifespan of UV-exposed parts, as confirmed in accelerated aging tests on polyolefins and ABS. In UL-94 tests, the combination allowed maintaining an LOI index above 28% in GF polyamides [93].
- TiO_2_ + HALS (Antagonistic): Titanium dioxide, used as a white pigment and opacifier in painted plastic parts like bumpers, exhibits photocatalytic activity under UV light. Its surface can deactivate HALS molecules by quenching their amine groups, thereby reducing their radical scavenging efficiency. This antagonism is particularly observed in high-pigment polyethylene or polypropylene formulations, where long-term UV stability is compromised despite the presence of HALS. Electron-optical studies have revealed oxidation of the amine groups in HALS in the presence of anatase TiO_2_, reducing their efficiency [99,100].
- Phosphorus-Based FR + Zinc Borate (Synergistic): In under-the-hood applications such as PA6-based engine covers, the combination of ammonium polyphosphate (APP) and zinc borate offers enhanced flame retardancy. Zinc borate contributes to char formation and suppresses smoke release. Molecularly, phosphorus compounds promote thermal dehydration, while borates form a protective ceramic-like layer. Together, they achieve a UL-94 V-0 rating with minimal compromise in mechanical strength.
- Plasticizer (DEHP) + Antioxidant (BHT) (Mixed): In flexible PVC used for automotive cable insulation or dashboards, di(2-ethylhexyl) phthalate (DEHP) increases polymer chain mobility, which can inadvertently accelerate the migration of small-molecule antioxidants like butylated hydroxytoluene (BHT). This results in elevated volatile organic compound (VOC) emissions and fogging in vehicle interiors. These effects have led to restrictions or replacements of such combinations in compliance with REACH and DIN 75201 standards.
- Compatibilizer (MA-g-PP) + Bio-Filler (Nanocellulose) (Antagonistic): In recyclable polymer blends (e.g., PP/PA used for interior panels), maleic anhydride-grafted polypropylene (MA-g-PP) may exhibit poor adhesion with hydrophilic fillers like nanocellulose. This is due to the lack of sufficient reactive compatibility between polar hydroxyl groups and nonpolar MA sites, resulting in phase separation and filler aggregation. The issue is often addressed by adding silane coupling agents or dual compatibilizers to improve dispersion and interfacial bonding.
- Organophosphates + Intumescent Flame Retardants (Mixed): In EV battery enclosures and electronic housings, combining organophosphate flame retardants with intumescent systems can enhance fire performance. However, these mixtures often suffer from poor matrix adhesion, particularly in nonpolar polymers like PP or PE. The incompatibility requires the use of reactive binders such as epoxy resins or silane-functionalized additives to stabilize the flame-retardant layer and ensure structural integrity under thermal stress.
- HALS + UV Pigments (e.g., Carbon Black) (Antagonistic): Carbon black is commonly used in black-colored ABS or polyolefin trims due to its UV-shielding properties. However, it also reduces UV penetration into the polymer matrix, thereby limiting the activation of HALS stabilizers. This can lead to insufficient stabilization in deeper layers of the polymer. Compensation through increased HALS dosage or selection of HALS variants less affected by pigment interaction is often necessary.
- ESO + Citrate Plasticizers (Synergistic): The combination of epoxidized soybean oil (ESO) and citrate esters in flexible PVC or TPU applications (e.g., cable sheaths, seals, interior soft pads) demonstrates synergistic behavior. Both additives reduce glass transition temperature and enhance flexibility while limiting migration. On a molecular level, their polar functional groups interact with the polymer chains through hydrogen bonding, forming a stable, low-VOC system compliant with automotive interior standards [101].

These observations underscore the complexity of additive design in automotive polymer engineering. While some interactions provide enhanced multifunctionality, others introduce compatibility challenges or trade-offs that must be addressed through careful formulation, modeling, and regulatory foresight. A mechanistic understanding of such interactions is essential for optimizing material performance and ensuring long-term durability under real-world conditions.

To better understand the mechanisms driving synergistic or antagonistic behavior, molecular-level analysis is essential. For instance, titanium dioxide exhibits photocatalytic activity due to its surface oxygen vacancies, which can interact with the nitrogen-containing functional groups of HALS, disrupting their radical scavenging efficiency. Similarly, the compatibility between organophosphate flame retardants and zinc borate is largely attributed to a dual mechanism of thermal dehydration (from phosphates) and glassy char formation (from borates), enhancing both flame retardancy and smoke suppression.

In contrast, the poor interaction between compatibilizers such as MA-g-PP and bio-fillers like nanocellulose stems from mismatched polarity. While MA-g-PP contains nonpolar polypropylene backbones and reactive maleic anhydride groups, nanocellulose surfaces are rich in hydroxyl groups, leading to insufficient interfacial bonding unless coupling agents are used.

The dual use of plasticizers and antioxidants, such as DEHP and BHT, introduces complex diffusion dynamics, where plasticizer-enhanced chain mobility accelerates the migration of low-molecular-weight stabilizers. This not only compromises oxidative stability but also raises concerns regarding volatile emissions and interior fogging in vehicles.

By incorporating a predictive modeling approach—based on Hansen solubility parameters or molecular dynamics simulations—future studies can anticipate such interactions and optimize formulations in silico before industrial validation.

While plasticizers such as HPCL offer low migration rates and high flexibility in PVC matrices, their industrial cost and limited availability may restrict scalability. Flame retardants, particularly halogenated systems, although effective, are increasingly regulated and incompatible with circular economy goals. UV stabilizers such as HALS are among the most effective for long-term stability, yet their performance may diminish in high-pigment or titanium dioxide-rich formulations. The transition from halogenated to halogen-free flame retardants is evident in automotive polymers, with phosphorus- and melamine-based systems offering effective fire protection while aligning with recyclability targets [102]. Incorporating nanoclays into polyamide matrices has shown synergistic improvements in flame retardancy and mechanical performance, as nanoparticle networks create a barrier effect that suppresses heat and mass transfer [103]. Thus, selecting an additive is not only a technical decision but one of trade-offs between performance, compliance, and sustainability. To illustrate how additive concentration impacts functional performance, a comparative study [95] examined the effect of varying zinc borate (ZB) concentrations on the flammability and mechanical properties of glass-fiber reinforced PA6 composites, Table 7.

The results indicate that increasing ZB content significantly improves flame resistance (LOI up by ~35%, UL-94 improved to V-0) but moderately reduces mechanical strength (~10% drop in tensile strength). This demonstrates the common trade-off in additive engineering, where functionality gains can compromise structural integrity. In line with recent advancements, a data-driven approach was employed to optimize additive selection for automotive polymer blends using a desirability function based on mechanical strength, thermal stability, and processing temperature. The model considered the following equation:

D = TS^α^ × PHRR^β^ × Tg^γ^ (1)

where D is the global desirability score (0–1), TS = tensile strength, PHRR = peak heat release rate, and Tg = glass transition temperature. α, β, γ are application-specific weights (e.g., β > γ for interior applications). The model predicted optimal formulations with 2.5 wt% HALS and 0.7 wt% phosphorus-based FRs for achieving a balance between UV resistance and flammability suppression in polypropylene-based dashboards.

Functional additive types, main roles, polymer compatibility, and challenges are presented in Table 8.

In order to offer a clear, comparative perspective on the performance and applicability of functional additives in automotive polymer matrices, Table 9 presents a synthesis matrix structured around seven industrially relevant criteria: polymer compatibility, enhanced properties, technical limitations, regulatory constraints, sustainability potential, and typical applications.

This synthesis matrix enables rapid comparison of additive options based on performance, limitations, and sustainability, supporting informed material selection in both automotive and related industries.

This comparative overview highlights the following important patterns:

- Firstly, HALS stabilizers remain the most widely compatible additives across polyolefins and engineering plastics, providing high UV resistance and oxidative stability. However, their interaction with pigments and relatively high cost limit their use in cost-sensitive applications. In contrast, organophosphates, although highly effective as flame retardants, raise concerns regarding migration and toxicity—especially under RoHS restrictions in the EU. Their partial substitution with intumescent systems is promising, yet not without processing and cost challenges.
- The use of bio-based plasticizers such as citrates and epoxidized soybean oil reflects the growing trend toward sustainable additives. Nevertheless, these options suffer from reduced thermal stability and still face limitations in high-performance environments like under-the-hood components. Similarly, anti-migration hybrid additives show potential for use in long-term contact applications (e.g., interior trims) but remain insufficiently studied in industrial-scale validations.
- From a recyclability and circularity perspective, compatibilizers such as maleic anhydride-grafted polymers provide enhanced interfacial adhesion in polymer blends. Yet, their impact on recyclability after multiple extrusion cycles is rarely addressed in literature, representing an ongoing research gap.
- Interestingly, functionalized nanoclays offer multifunctional benefits, such as mechanical reinforcement and improved barrier properties, but pose dispersion and cost-related challenges, especially in large-scale automotive manufacturing.
- As summarized in Table 8, the trade-offs between flame retardant efficiency, cost, and regulatory status become particularly evident in the case of organophosphates versus intumescent systems. While several fossil-based additives continue to dominate due to maturity and proven effectiveness, bio-based or hybrid alternatives are increasingly being integrated—albeit at a slower pace and with unresolved limitations. This landscape calls for deeper multidisciplinary research and the development of selection tools adapted to specific applications and processing constraints.

Case Study 1—UV Stability in Exterior Automotive Trims

In polypropylene/ABS mirror housings, the synergistic combination of HALS 770 (1 wt%) and benzotriazole (0.8 wt%) reduced ΔE color change by 80% and retained 92% tensile strength after 2000 h of ISO 4892 accelerated UV exposure, compared to single-additive systems. This performance improvement is consistent with the findings [104], which demonstrated that HALS-benzotriazole systems offer superior long-term stability by addressing both photoinitiation and radical propagation steps in polymer degradation. Similarly, recent studies confirm that incorporating HALS into polyolefins significantly enhances UV resistance and mechanical property retention under prolonged exposure. BMW has introduced phthalate-free plasticized PVC in door panels and dashboards since 2015, using terephthalate-based and citrate plasticizers to reduce VOC emissions in vehicle interiors [105].

Case Study 2—Flame Retardancy in Engine Covers

Polyamide 6 engine covers formulated with 15 wt% ammonium polyphosphate (APP) and 10 wt% zinc borates achieved a UL-94 V-0 rating, reduced smoke density by 25%, and maintained tensile strength above 90% of the baseline. This result aligns with the synergistic flame-retardant behavior reported by, where APP promoted char formation while zinc borate contributed to smoke suppression and thermal insulation. The combined mechanism provides both improved fire safety and compliance with automotive regulatory requirements. Toyota has implemented halogen-free flame-retardant polyamide for wire harness connectors, relying on red phosphorus and metal hydroxides to comply with both fire safety and recyclability requirements [106]

Case Study 3—Low-VOC Cable Insulation

PVC cable insulation plasticized with 20 phr acetyl tributyl citrate (ATBC) exhibited 35% lower migration and 25% lower VOC emissions (DIN 75201) compared to DEHP-plasticized equivalents, without significant loss in mechanical properties. To [107] demonstrated that non-phthalate plasticizers such as TOTM and ATBC can achieve markedly lower migration rates—up to 350 times less than DEHP—while maintaining flexibility. Non-phthalate plasticizers such as adipates and terephthalates have been increasingly adopted due to their lower toxicity and compliance with REACH and RoHS directives, though their thermal stability remains a limitation [108]. Moreover, ref. [109] observed that plasticizers such as DOTP and DINCH have lower diffusion coefficients and reduced mass loss compared to traditional phthalates, supporting their adoption for low-emission automotive interior applications. Volkswagen applied HALS-based UV stabilizers in polypropylene bumpers and exterior trims, significantly reducing surface chalking and gloss loss after accelerated weathering tests. Ford employs hindered phenol/phosphite antioxidant blends in polypropylene radiator end tanks and intake manifolds, improving long-term heat resistance in under-the-hood components [110,111,112].

## 5. Future Research Directions

To address the absence of standardized evaluation procedures, we propose a draft testing framework for functional additives in automotive polymers, consisting of the following:

- Limiting Oxygen Index (LOI) and UL-94 flammability testing;
- Thermogravimetric analysis (TGA) for thermal stability;
- UV resistance testing per ISO 4892;
- Accelerated migration testing under cyclic temperature/humidity;
- Recyclability assessment using both mechanical reprocessing and chemical depolymerization metrics.

This minimum set of performance indicators can serve as a baseline for comparative research and industrial benchmarking. Despite the progress made in the development and integration of functional additives in polymer matrices for automotive applications, several critical areas remain underexplored or insufficiently addressed. Future research should focus on the following five strategic directions:

- Standardization of Testing Methodologies—Current research employs diverse testing protocols for thermal stability, flame retardancy, migration behavior, and mechanical performance. This lack of uniformity prevents meaningful comparisons across studies and hinders reproducibility. Developing and adopting internationally harmonized testing standards, particularly under conditions simulating automotive environments (e.g., high humidity, cyclic thermal stress, UV exposure), is essential to ensure the reliability and scalability of laboratory findings.
- Long-Term Durability and Recyclability Assessment—Although many additives show promising short-term performance, few studies assess their impact on the recyclability and aging behavior of polymer matrices. Future investigations should explore how additives (especially compatibilizers and stabilizers) influence material degradation during multiple extrusion cycles, oxidative aging, and recycling under mechanical, thermal, and chemical stressors. Such work is critical in the context of the Circular Economy Action Plan and extended producer responsibility regulations.
- Development of Bio-Based, Thermally Stable Additives—A major challenge for sustainable materials science is the limited availability of bio-based additives with high thermal and chemical stability. Research should prioritize the synthesis and characterization of bio-derived flame retardants, plasticizers, and stabilizers that can match or exceed the performance of fossil-based analogs, particularly in high-temperature and high-shear automotive applications (e.g., EV drivetrains, battery housings).
- AI-Driven Additive Selection and Performance Prediction-Artificial intelligence and machine learning algorithms can significantly accelerate the discovery and optimization of additive polymer systems. Future studies should aim to create open-access databases and predictive models that correlate molecular structure, additive loading, processing conditions, and functional performance. Such tools could support rapid prototyping and reduce dependency on trial-and-error experimentation.
- Regulatory-Integrated Design Frameworks—Future work should integrate regulatory foresight into additive design. This involves assessing the long-term viability of additives in light of evolving restrictions (REACH, RoHS, ELV) and incorporating Life Cycle Assessment (LCA) criteria early in development. Research that combines material science with regulatory and economic modeling will better position new additives for industrial adoption and compliance.

**Figure 8:**
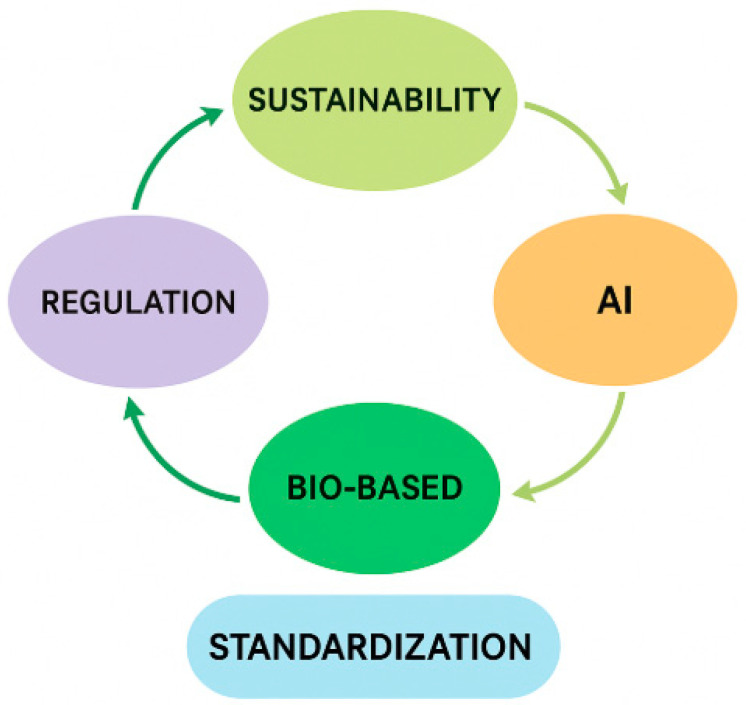
Conceptual integration of these future directions.

These five strategic research areas—standardization, long-term durability and recyclability, development of bio-based additives, AI-driven additive selection, and regulatory-integrated design—are not isolated initiatives but interconnected dimensions of the same innovation framework. Figure 8 illustrates the conceptual integration of these future directions as part of a cohesive roadmap for advancing functional additive design in automotive polymer systems. Each element—standardization, sustainability, artificial intelligence (AI), bio-based innovation, and regulatory frameworks—acts both as a driver and as a dependent variable within the systemic evolution of polymer technology. The roadmap underscores that no single dimension can evolve in isolation; rather, progress depends on mutual reinforcement. For instance, AI enables predictive modeling and real-time process optimization but requires robust data standards and regulatory alignment. Similarly, the adoption of bio-based materials hinges on both regulatory incentives and the ability to meet standardized performance metrics. This interconnectivity forms the backbone of a resilient and future-ready polymeric design ecosystem tailored to automotive industry demands.

While the primary focus of this review is automotive applications, the methodologies, synthesis tables, and selection frameworks presented are readily transferable to other high-performance polymer sectors. For example, flame-retardant–stabilizer synergies identified herein are relevant to aerospace interiors; low-VOC plasticizer systems apply to electronics casings; and nanofiller-enhanced UV stabilizers could benefit construction materials exposed to high UV flux. Incorporating AI-guided selection tools, targeted life-cycle analyses for emerging bio-based additives, and accelerated multi-factor environmental testing will further extend the applicability of these findings across industries. We propose a draft testing protocol integrating the following:

- Development of an AI-guided additive selection tool trained on multi-sector performance databases.
- Design of a standardized testing protocol combining LOI, TGA, and recyclability assessments.
- Implementation of targeted LCA studies for bio-based additives in high-demand sectors such as aerospace and electronics.

## 6. Conclusions

Functional additives represent a cornerstone in the design and engineering of polymeric materials for the automotive sector. This review has emphasized not only their role in enhancing the mechanical, thermal, and chemical resistance of polymers, but also the need for careful consideration of additive–polymer compatibility, environmental safety, and processing efficiency.

The present review provides a comprehensive and critical overview of functional additives used in polymer matrices for automotive applications, emphasizing their compatibility, performance, limitations, and regulatory challenges. Based on a systematic analysis of recent literature and industrial trends, the following conclusions can be drawn:

- Functional additives remain essential for enhancing the thermal, mechanical, and flame-retardant properties of polymers in automotive settings. However, their selection must balance multiple factors, including material compatibility, toxicity, processing ease, and end-of-life considerations.
- Bio-based and hybrid additives show growing potential in aligning performance with sustainability goals. Nonetheless, their adoption is hindered by lower thermal stability and limited validation under realistic processing and service conditions.
- The comparative synthesis presented in Table 8 underlines the current trade-offs between functionality, safety, regulatory acceptance, and environmental impact. Such trade-offs must be navigated through multidisciplinary approaches that bridge material science, regulatory policy, and industrial engineering.
- Research gaps remain substantial, particularly in the areas of recyclability, aging behavior, and additive–matrix interaction mechanisms. Greater attention is needed toward standardized testing, long-term performance data, and predictive modeling.
- Future progress will rely on integrated innovation, combining eco-design principles with AI-guided material selection, life cycle analysis, and cross-sectoral collaboration between academia, regulatory bodies, and the automotive industry.

While the individual roles of plasticizers, flame retardants, UV stabilizers, and antioxidants are well-documented, this work underlines the necessity of integrating multiple selection criteria: performance under service conditions, regulatory compliance, long-term stability, and industrial feasibility. Our structured approach—guided by four core research questions—has enabled a functional mapping of additive types to specific challenges in automotive applications.

Key findings include the following:

- UV stabilizers such as HALS and benzotriazole derivatives are essential for extending the life of exposed components, but their interaction with polymer type and geometry must be carefully evaluated.
- Plasticizers, especially in PVC systems, require precise dosing and compatibility validation to avoid additive migration and environmental hazards.
- Flame retardants must balance fire performance with toxicity profiles and recyclability, a major concern in current sustainability frameworks.
- Additives also play a critical role in processing efficiency, reducing energy loss, mold friction, and thermal degradation during polymer transformation steps.
- Despite significant advancements, knowledge gaps remain regarding the synergistic or antagonistic effects between multiple additives, especially under combined stress conditions (e.g., heat + UV + mechanical load). To address these challenges, we propose several areas for further investigation, as follows:
- Development of standardized compatibility assessment protocols for additive–polymer systems.
- In-depth study of multifunctional additive systems that combine flame retardancy, UV stability, and processability in a single compound.
- Increased focus on bio-based and low-toxicity additives, aligned with circular economy goals and upcoming regulatory frameworks.
- Integration of machine learning tools for additive selection and predictive modeling of material performance.
- This review serves as a foundation for engineers, material scientists, and product developers to make informed decisions in the design of next-generation polymeric materials for automotive use, combining performance, sustainability, and manufacturability.
- Based on the comparative analysis, the following recommendations are proposed:
- For researchers: Prioritize synergistic additive systems that deliver multifunctional performance without overloading the matrix. Integrate predictive modeling early in formulation.
- For industry/OEMs: Select additives within optimal loading windows to avoid mechanical compromise. Implement in-service simulation testing for migration and durability.
- For policymakers: Encourage standardization of additive testing protocols and incentivize adoption of bio-based additives with proven thermal stability.
- Key trade-offs include balancing flame retardancy with tensile strength, UV stability with pigment compatibility, and bio-based sustainability with cost and thermal limits.

This review has highlighted the critical role of plasticizers, flame retardants, UV stabilizers, and antioxidants in enhancing the performance and safety of automotive polymers. Despite the significant advances, trade-offs between functionality, cost, and sustainability remain evident. Future research should prioritize three directions: (i) the development of synergistic additive systems combining bio-based and conventional stabilizers to balance performance with eco-efficiency; (ii) improving recyclability and end-of-life treatment by designing additives compatible with circular economy strategies; and (iii) expanding industrial-scale validation through pilot projects in automotive applications. Addressing these challenges will be essential to achieve durable, safe, and environmentally responsible polymer solutions for the next generation of vehicles.

## Figures and Tables

**Table 1:** Examples of polymer components and materials used in automotive systems.

Plastic Components	Types of Polymers
Bumper	PS, ABS, PC/PBT
Chairs	PUR, PP, PVC, ABS, PA
Dashboard	PP, ABS, SMA, PPE, PC
Power system	HDPE, POM, PA, PP, PBT
Body	PP, PPE, UP
Components under the hood	PA, PP, PBT
Interior decoration	PP, ABS, PET, POM, PVC
Electrical components	PP, PE, PBT, PA, PVC
Exterior decoration	ABS, PA, PBT, POM, ASA, PP
light	PC, PBT, ABS, PMMA, UP
Upholsterer’s	PVC, PUR, PP, PE
Liquid tanks	PP, PE, PA

**Table 2:** Common phthalate plasticizers and their applications.

Phthalates	Abbreviation	Applications
Dibutyl phthalate	DBP	PVC, PVA, rubber
Diethylhexyl phthalate	DEHP	dolls, shoes, raincoats, clothing, medical devices, plastic tubing and intravenous storage bags
Diisononyl phthalate	DINP	PVC (balls, spoons, toys, gloves, drinking straws)
Diisodecyl phthalate	DIDP	PVC (leather for car interiors and PVC floors)
Butyl benzyl phthalate	BBP	PVC, polyurethane, polysulfide (vinyl flooring, sealants, adhesives, car care products, automotive trim, food conveyor belts, food packaging material)

**Table 3:** Summary of functional additives and their effects on polymer matrices.

Additive Type	Functional Effect	Compatible Polymers	Effects on Matrix
Plasticizers	Increase flexibility, reducing Tg	PVC, PVA	Increased flexibility
Flame retardants	Delay ignition	PA, PP, PC, PBT	Fire safety, reduced smoke emission
UV stabilizers	UV radiation protection	PS, PP, PVC, PC	Preventing yellowing
Antioxidants	Inhibit oxidation	PA, PE, rubbers	Preventing thermal degradation, increased durability
Lubricants/processing aids	Reduce viscosity, improve processing	All thermoplastics	Energy savings, improved protection
Fillers	Reinforcement, reduce costs	PP, PE, PVC	Increased resilience

**Table 4:** The functions, compatibility, applications, and main risks associated with functional additives used in the automotive industry.

Additive Type	Main Function	Polymer Compatibility	Typical Automotive Applications	Typical Loading (% wt)	Challenges/Notes
Plasticizers (PAE, NPP)	Increases flexibility, lowers Tg	PVC, PVA	Cable insulation, interior trims, flexible seals	5–30%	Migration risk; endocrine disruption; many PAE phased out in EU/US
Flame retardants (PFRs)	Reduces flammability, delays ignition	PA, PP, PBT, PC	Engine covers, under-hood parts, dashboards	10–25%	Toxicity concerns; halogen-free systems gaining ground for recyclability
UV stabilizers (HALS, BZT, UVF)	Prevents photodegradation, discoloration	PS, PP, PVC, PC, ABS	Exterior trims, bumpers, headlights	0.1–2%	Efficiency depends on exposure intensity, wall thickness, and pigment load
Antioxidants (SDPA, phenolics)	Inhibits oxidation, extends lifespan	PA, PE, PP, rubbers	Fuel systems, gaskets, under-hood components	0.3–1%	Degrade at prolonged high heat; sometimes require synergists (e.g., thioesters)
Lubricants/Processing aids	Improves processability, reduces viscosity	Most thermoplastics	Injection/extrusion of body panels, interiors	0.2–2%	Overdosing may reduce mechanical strength or surface adhesion
Fillers (talc, CaCO_3_, silica)	Improves rigidity, reduces cost	PP, PE, PVC	Dashboards, body panels, door modules	10–40%	Can reduce impact strength; affects moldability at high loadings

**Table 5:** Quantitative Comparison of Additive Performance.

Additive	Used Concentration (%)	Targeted Property	Initial Value	Value Obtained	Reference
Alumina Trihydrate (ATH)	5	Flame retardancy	PHRR: 430 kW/m^2^	PHRR: 225 kW/m^2^	[67]
Zinc Borate	10	Flame spread delay	LOI: 18%	LOI: 28%	[68]
Melamine Cyanurate	15	Self-extinguishing improvement	UL94: V-2	UL94: V-0	[69]
HALS (Hindered Amine Light Stabilizer)	2	UV stability	ΔE > 6 after 300 h UV	ΔE < 2 after 300 h UV	[70]
BHT (Butylated Hydroxytoluene)	1	Oxidative stability	High peroxide after 100 h	Peroxide reduced by 40%	[71]

**Table 6:** Interactions between additives.

Additive Combination	Synergistic/Antagonistic	Effect on Performance	Observations	Observed Interactions and Molecular Explanation
HALS + BZT	Synergistic	Increased UV stability	Optimal in PP, ABS systems	Synergistic. HALS (Hindered Amine Light Stabilizers) neutralize radicals formed during polymer degradation, while BZT acts earlier in the degradation pathway by absorbing UV radiation. Their combined use addresses both photoinitiation and radical propagation, leading to improved photostability. Molecular synergy is enhanced when both additives are properly dispersed and stabilized within the polymer matrix.
TiO_2_ + HALS	Antagonistic	HALS activity reduced	Titanium dioxide may quench HALS reactivity	Antagonistic. Titanium dioxide, especially in anatase form, can act as a photocatalyst under UV exposure, generating hydroxyl radicals. These radicals oxidize the amine functional groups of HALS, diminishing their radical-scavenging activity. This interaction results in reduced efficiency of HALS and accelerated degradation in high TiO_2_-content systems.
Phosphorus FR + Zinc Borate	Synergistic	Improved flame retardancy	Common in PA6	Synergistic. Phosphorus compounds promote the formation of a protective char layer during combustion, while zinc borate enhances the intumescent structure and contributes to smoke suppression. The thermal decomposition of zinc borate also releases water, providing a cooling effect. Together, they form a stable, glassy residue that improves flame retardancy and reduces toxicity.
Plasticizer + Antioxidant	Mixed	May accelerate migration	Depends on molecular weight	Limited compatibility. Maleic anhydride-grafted polypropylene provides polar groups that can interact with hydrophilic fillers like nanocellulose, but dispersion and adhesion remain suboptimal unless coupling agents are introduced. Interfacial bonding is often weak due to polarity mismatch, and molecular mobility of the compatibilizer is insufficient to ensure uniform encapsulation of cellulose fibrils.

**Table 7:** Additive concentration impacts functional performance.

ZB Content (wt%)	LOI (%)	UL-94 Rating	Tensile Strength (MPa)	Elongation (%)
0	21.5	V-2	84.2	2.3
5	26.7	V-0	79.8	2.0
10	29.1	V-0	76.1	1.8

**Table 8:** Functional additive types, main roles, polymer compatibility, and challenges.

Additive Type	Main Function	Polymer Compatibility	Typical Automotive Applications	Dosage	Challenges/Notes	Evidence Strength
Plasticizers (PAE, NPP)	Increases flexibility, lowers Tg	PVC, PVA	Cable insulation, interior trims, flexible seals	5–30%	Migration risk; endocrine disruption; many PAE phased out in EU/US	**Moderate**—industrial use, but regulatory phase-out in multiple regions.
Flame retardants (PFRs)	Reduces flammability, delays ignition	PA, PP, PBT, PC	Engine covers, under-hood parts, dashboards	10–25%	Toxicity concerns; halogen-free systems gaining ground for recyclability	**High**—standardized testing (UL 94, LOI) and wide industrial application.
UV stabilizers (HALS, BZT, UVF)	Prevents photodegradation, discoloration	PS, PP, PVC, PC, ABS	Exterior trims, bumpers, headlights	0.1–2%	Efficiency depends on exposure intensity, wall thickness, and pigment load	**High**—ISO 4892 compliance, validated in automotive field use.
Antioxidants (SDPA, phenolics)	Inhibits oxidation, extends lifespan	PA, PE, PP, rubbers	Fuel systems, gaskets, under-hood components	0.3–1%	Degrade at prolonged high heat; sometimes require synergists (e.g., thioesters)	**High**—proven in long-term thermal aging and oxidation studies.
Lubricants/Processing aids	Improves processability, reduces viscosity	Most thermoplastics	Injection/extrusion of body panels, interiors	0.2–2%	Overdosing may reduce mechanical strength or surface adhesion	**Moderate**—widely applied, but performance strongly formulation-dependent.
Fillers (talc, CaCO_3_, silica)	Improves rigidity, reduces cost	PP, PE, PVC	Dashboards, body panels, door modules	10–40%	Can reduce impact strength; affects moldability at high loadings	**High**—full-scale automotive validation, extensive literature evidence.

**Table 9:** Functional additives, improved properties, limitations, and sustainability.

Functional Additive	Compatible Polymers	Improved Properties	Technical Limitations	Regulatory Risks	Sustainability	Typical Applications	Evidence Strength
HALS (light-thermal stabilizers)	PP, PE, PA, ABS	UV resistance, aging stability	High cost, pigment interactions	REACH—partially restricted	Fossil-based	Exterior parts, colored interior components	**High**—ISO 4892 compliance, OEM validation.
Organophosphates (flame retardants)	PVC, PA, PC, TPU	UL94 V-0 rating, smoke reduction	Migration potential, toxicity	RoHS-restricted	Fossil-based	Electrical connectors, housings, under-the-hood parts	**High**—standardized flame tests, wide industrial use.
Phenolic + phosphite stabilizers	PE, PP, TPO	Oxidative protection during processing	Moisture sensitivity	REACH-approved	Fossil-based	Injection-molded parts, recycled compounds	**Moderate**—common industrial additive, limited long-term environmental data.
Compatibilizers (MA-g-PP/SEBS-g-MA)	PP/PA, PC/ABS, PP/PE	Interfacial adhesion, toughness	Potential recyclability impact	Not restricted	Fossil-based	Blends, housings, structural components	**Moderate**—industrial relevance, but recyclability impact under-researched.
Bio-plasticizers (citrates, ESO)	PVC, TPU	Flexibility, low glass transition temp.	Low thermal stability	Mostly approved	Bio-based	Seals, cables, soft pads	**Moderate**—good lab/industrial data, thermal stability limits.
Intumescent systems (APP + melamine)	PA, PE, EVA	Char barrier formation, fire protection	Poor matrix adhesion, high cost	EU-approved	Partially bio-based	Electrical boards, EV components	**High**—validated under EN fire testing standards.
Functionalized nanoclays	PP, PA, PBT	Stiffness, barrier properties, flame delay	Dispersion challenges, cost	Acceptable	Fossil-based	Fuel tanks, HVAC components	**Moderate**—lab and pilot-scale validation, cost barriers remain.
Anti-migration additives (organic hybrids)	PVC, TPE	Reduced plasticizer migration	Limited data, higher cost	Under evaluation	Partially bio-based	Automotive interiors, extended contact applications	**Low**—limited peer-reviewed data, mostly preliminary studies.

## Data Availability

The original contributions presented in this study are included in the article. Further inquiries can be directed to the corresponding author.

## Abbreviations

**ABS**	Acrylonitrile Butadiene Styrene
**ASA**	Acrylonitrile–Styrene–Acrylate
**BBP**	Butyl benzyl phthalate
**BZTUV**	Benzotriazole
**DBP**	Dibutyl phthalate
**DEHP**	Diethylhexyl phthalate
**DIDP**	Diisodecyl phthalate
**DINP**	Diisononyl phthalate
**HALS**	Light stabilizers based on sterically hindered amines
**HDPE**	High-density polyethylene
**HPCL**	Hyperbranched poly(ε-caprolactone)
**NPP**	Non-phthalates
**PA**	Polyamide
**PAE**	Phthalates
**PBT**	Polybutylene terephthalate
**PC**	Polycarbonate
PE	Polyethylene
PET	Polyethylene terephthalate
PFR	Flame retardants
PMMA	Polymethylmethacrylate
POM	polyoxymethylene
PP	Polypropylene
PPE	Polyphenylene ether
PS	polystyrene
PUR	Polyurethane
PVA	Polyvinyl alcohol
PVC	Polyvinyl chloride
SDPA	Substituted diphenylamine antioxidants
SMA	Styrene Maleic Anhydride
UP	Unsaturated polyester
UVF	UV filters
UV	Ultraviolet
